# Exploration of the correlation between clinical indicators and prognosis in hospitalized children with pneumonia and construction of a risk prediction model based on machine learning algorithms

**DOI:** 10.3389/fmed.2026.1747935

**Published:** 2026-01-28

**Authors:** Jin Xue, Guangzhong He, Qiaoying Chen

**Affiliations:** School of Medicine, Shaanxi University of International Trade and Commerce, Xianyang, China

**Keywords:** childhood pneumonia, clinical indicators, machine learning, prognosis, risk prediction model, single-center study

## Abstract

**Background:**

Childhood pneumonia is a leading cause of hospitalization and death in children under 5 years globally. Its prognosis varies individually and is affected by multiple clinical indicators, while traditional assessment lacks quantitative risk stratification tools. Machine learning (ML) enables comprehensive analysis of high-dimensional clinical data, making it valuable for identifying key prognostic factors and building robust prediction models to optimize clinical decision-making.

**Methods:**

A total of 582 hospitalized children (1 month–5 years) with community-acquired pneumonia were retrospectively enrolled (January 2022–June 2025). Demographic, laboratory (WBC, CRP, PCT, LYM%, serum albumin), vital sign, and underlying disease data were collected. Adverse prognosis was defined as a composite of prolonged hospitalization (>7 days), PICU admission, or in-hospital death. Patients were randomly split into training (*n* = 407) and validation (*n* = 175) sets (7:3). XGBoost, Random Forest (RF), and Logistic Regression (LR) models were constructed, with performance evaluated by AUC, accuracy, sensitivity, and specificity. Class imbalance was addressed using stratified random sampling during dataset splitting to maintain consistent adverse prognosis rates between training and validation sets. SHAP values analyzed indicator importance. Missing data (all < 5%) were imputed via mean imputation; a sensitivity analysis comparing mean imputation with multiple imputation confirmed no significant impact on model performance.

**Results:**

Adverse prognosis occurred in 121 (20.8%) children. The XGBoost model outperformed RF and LR, with validation-set AUC 0.84 (95% CI: 0.78∼0.90), accuracy 81.1%, sensitivity 78.6%, and specificity 82.3%. Model calibration was verified via Hosmer-Lemeshow test (*p* = 0.312), indicating good agreement between predicted and observed risks. Top 5 key indicators were admission PCT, CRP, respiratory rate, age < 6 months, and blood oxygen saturation. PCT > 2 ng/mL (OR = 3.95) and CRP > 40 mg/L (OR = 3.52) significantly increased adverse prognosis risk. Etiological data (viral, bacterial, mixed infection) were unavailable in 41.2% (240/582) of cases; among available data (342/582), 58.5% (200/342) were viral (including 12 cases of COVID-19), 32.2% (110/342) bacterial, and 9.3% (32/342) mixed infections. Sensitivity analysis excluding COVID-19 cases (*n* = 12) showed no substantial change in model performance (AUC = 0.83, 95% CI: 0.77∼0.89).

**Conclusion:**

The XGBoost-based model effectively identifies high-risk children with pneumonia, with PCT, CRP, and respiratory rate as key predictors. It provides a practical tool for clinical risk stratification and personalized management. The model’s cutoffs for PCT (>2 ng/mL) and CRP (>40 mg/L) align with existing pediatric pneumonia predictive scores (e.g., PRIEST score) but offer improved discriminative power by integrating multi-dimensional indicators and ML-driven interactions.

## 1 Introduction

Childhood pneumonia is the leading infectious cause of hospitalization and death among children under 5 years old worldwide (1). According to the World Health Organization (WHO), approximately 142 million children sought medical care for pneumonia globally in 2022, with over 2 million requiring hospitalization and 294,000 deaths reported. These deaths account for 16% of all deaths in children under 5. In China, the hospitalization rate for childhood pneumonia accounts for 20–30% of total pediatric admissions, with community-acquired pneumonia (CAP) representing more than 80% of cases. Some critically ill children may progress to severe complications such as respiratory failure and sepsis, and even develop long-term sequelae including impaired lung function and neurodevelopmental disorders (2, 3).

The prognosis of childhood pneumonia is influenced by multiple factors. Infants younger than 6 months are prone to disease progression after infection due to immature immune systems (4). Laboratory indicators such as procalcitonin (PCT) and C-reactive protein (CRP) reflect the severity of infection, and elevated levels often indicate bacterial infection and increased risk of adverse prognosis (5). Abnormal vital signs, such as increased respiratory rate and low blood oxygen saturation, are directly associated with impaired pulmonary ventilation and serve as core indicators for judging disease severity (6). Underlying conditions including congenital heart disease and preterm birth history further increase the risk of complications following infection in affected children (7). However, traditional prognostic assessment relies heavily on the subjective experience of clinicians, who often judge disease severity based on single indicators such as body temperature or white blood cell count. This approach struggles to integrate multi-dimensional data for quantitative risk stratification, leading to potential underidentification of high-risk children or overtreatment of low-risk patients (8).

Machine learning (ML), a core technology of artificial intelligence, can automatically explore potential correlations in high-dimensional data through algorithms. It has been widely applied in the medical field for disease diagnosis, prognostic prediction, and treatment optimization (9, 10). The Extreme Gradient Boosting (XGBoost) algorithm reduces the risk of model overfitting by integrating multiple decision trees, demonstrating high accuracy and robustness in clinical risk prediction (11). The Random Forest (RF) algorithm effectively handles nonlinear data and is suitable for analyzing interactions between multiple indicators (12). Although Logistic Regression (LR) is a traditional statistical method, it is often used as a benchmark for ML models due to its interpretability (13). Currently, ML has been applied in prognostic assessment of respiratory diseases such as adult pneumonia and childhood asthma, but studies focusing on hospitalized children with pneumonia still face limitations including small sample sizes, single-dimensional indicators, and insufficient model validation (14, 15). Methodological rigor in clinical ML studies, including measurement reliability of physiological markers and validity of data imputation, is critical for ensuring model generalizability.

This study enrolled 582 hospitalized children with pneumonia, integrating multi-dimensional data including demographic characteristics, laboratory indicators, vital signs, and underlying diseases. Three algorithms—XGBoost, RF, and LR—were used to construct prognostic risk prediction models. Model performance was evaluated using objective indicators, and key prognostic factors were identified. The aim of this study is to provide a precise tool for clinical risk stratification and personalized diagnosis and treatment of childhood pneumonia, ultimately improving patient prognosis and reducing the waste of medical resources. The model’s generalizability is limited to similar tertiary hospital settings in Shaanxi Province; external validation across diverse healthcare contexts (e.g., primary hospitals, other provinces) is needed to confirm broader applicability.

## 2 Materials and methods

### 2.1 Study participants

This retrospective study enrolled children with pneumonia who were hospitalized in the Department of Respiratory Medicine from January 2022 to June 2025. Inclusion criteria were as follows: age between 1 month and 5 years; compliance with the diagnostic criteria outlined in the Guidelines for the management of community-acquired pneumonia in children (2024 revision) (16), which require symptoms such as cough and fever (body temperature ≥ 38.5°C) combined with chest imaging (chest X-ray or CT) showing pneumonic signs including lobar consolidation and patchy opacities; hospitalization duration of at least 24 h; and complete clinical data including laboratory tests, vital sign records, and prognostic information. Exclusion criteria included hospital-acquired pneumonia (onset ≥ 48 h after admission); comorbidity with other lung diseases such as tuberculosis, lung tumors, or cystic fibrosis; missing data for ≥ 2 key indicators; and interrupted prognostic follow-up due to transfer to another hospital or voluntary discharge. Adverse prognosis was defined as a composite of prolonged hospitalization (>7 days), PICU admission, or in-hospital death. Prolonged hospitalization (>7 days) was selected based on the average 5–7-day hospital stay for uncomplicated CAP specified in the Clinical Guidelines for the Diagnosis and Treatment of Childhood Pneumonia, with > 7 days indicating persistent inflammation, incomplete pathogen clearance, or developing complications that require extended management.

A total of 582 children were finally included, comprising 326 males (56.0%) and 256 females (44.0%). Ages ranged from 1 month to 5 years, with a median age of 18 months and an interquartile range of 10–28 months. Regarding underlying diseases, 32 cases (5.5%) had congenital heart disease, 28 cases (4.8%) had a history of preterm birth, and 522 cases (89.7%) had no underlying conditions. This study was approved by the hospital’s Ethics Committee (Ethics No. 2022038). As a retrospective study, informed consent was waived, but all data were anonymized in compliance with the ethical requirements of the Declaration of Helsinki.

From July 2025 to October 2025, 186 children with community-acquired pneumonia hospitalized in the Department of Respiratory Medicine of 2 other tertiary hospitals in Shaanxi Province (Xianyang Central Hospital and Baoji People’s Hospital) were enrolled as the external validation cohort, with inclusion and exclusion criteria identical to those of the single-center cohort. The external validation cohort consisted of 186 children, including 104 males (55.9%) and 82 females (44.1%), with a median age of 17 months (IQR: 9–26 months). The incidence of adverse prognosis was 21.5% (40/186), including 31 cases of prolonged hospitalization, 8 cases of PICU admission, and 1 case of in-hospital death.

### 2.2 Data collection

Clinical data of the children were extracted from the hospital’s Electronic Medical Record System (EMRS) and categorized into demographic characteristics, laboratory indicators, vital signs, and underlying diseases. The specific definitions and detection methods of the indicators are described below.

Demographic characteristics included age (accurate to months) and gender. Based on clinical practice, age was divided into four subgroups: < 6 months, 6 months to 1 year, 1–3 years, and 3–5 years to analyze the impact of different age groups on prognosis.

For laboratory indicators, venous blood was collected from all children within 24 h of admission, and the following parameters were detected using an automatic biochemical analyzer (model: Beckman AU5800). White blood cell (WBC) count had a normal reference range of 4–12 × 10^9^/L, with elevation indicating infection. C-reactive protein (CRP) was measured by immunoturbidimetry, with a normal reference value < 8 mg/L and values > 40 mg/L suggesting severe infection. Procalcitonin (PCT) was detected by chemiluminescence, with a normal reference value < 0.5 ng/mL and values > 2 ng/mL indicating sepsis risk. Lymphocyte percentage (LYM%) had a normal reference range of 20–40%, with reduction reflecting immune suppression. Serum albumin (ALB) was measured by bromocresol green method, with a normal reference range of 35–50 g/L and reduction indicating malnutrition.

Vital signs were recorded using standardized equipment upon admission. Body temperature was measured axillary, with ≥ 38.5°C defined as high fever. Respiratory rate (RR) was considered abnormal according to age-specific standards: > 50 breaths per minute for children < 1 year and > 40 breaths per minute for those 1–5 years old. Oxygen saturation (SpO2 ) was detected by pulse oximetry, with < 94% defined as hypoxemia.

Underlying diseases were extracted from the “past medical history” field in the medical records, including congenital heart disease (such as ventricular septal defect and atrial septal defect) and preterm birth history (birth at a gestational age < 37 weeks).

### 2.3 Definition of outcome indicator

The primary outcome was defined as adverse prognosis, a composite endpoint including prolonged hospitalization, admission to the Pediatric Intensive Care Unit (PICU), and in-hospital death. The rationale for combining these outcomes is their shared association with disease severity and the need for enhanced clinical intervention. Prolonged hospitalization reflects unresolved infection or complications; PICU admission indicates life-threatening organ dysfunction; and in-hospital death represents the most severe outcome. A sensitivity analysis using alternative definitions (e.g., excluding prolonged hospitalization) showed consistent model performance (AUC = 0.82, 95% CI: 0.76∼0.88) (Supplementary Table S1). Prolonged hospitalization was defined as a total hospital stay > 7 days, referencing the average hospitalization duration of 5–7 days for CAP specified in the Clinical Guidelines for the Diagnosis and Treatment of Childhood Pneumonia. PICU admission was required for children with respiratory failure, sepsis, or other conditions necessitating tracheal intubation and vasoactive drug therapy. In-hospital death referred to death due to pneumonia and its complications during hospitalization.

### 2.4 Model construction and validation

#### 2.4.1 Data preprocessing

Missing data were handled using mean imputation, with the missing rate of all indicators below 5%. A sensitivity analysis comparing mean imputation with multiple imputation (5 iterations) was performed, showing no significant differences in model AUC (mean imputation: 0.84 vs. multiple imputation: 0.83; *p* = 0.612) (Supplementary Table S2). Continuous variables such as white blood cell count, C-reactive protein, and respiratory rate were normalized via Z-score standardization to eliminate the impact of dimensional differences on model training. Categorical variables including gender and underlying diseases were converted into dummy variables, with 0 representing absence and 1 representing presence.

Participants were divided into a training set (*n* = 407) and an internal validation set (*n* = 175) at a 7:3 ratio using stratified random sampling to maintain the same adverse prognosis rate (20.8%) across sets, ensuring no significant differences in age, gender, underlying diseases, or incidence of adverse prognosis among the groups (*P* > 0.05). Detailed grouping information is presented in Table 1. Meanwhile, 186 cases from the multicenter external validation cohort were included for independent validation.

**TABLE 1:** Comparison of baseline characteristics between the training set and validation set.

Characteristics	Total population (*n* = 582)	Training set (*n* = 407)	Validation set (*n* = 175)	Test statistic	*P*- value
Gender (male), n (%)	326 (56.0%)	228 (56.0%)	98 (56.0%)	χ^2^ = 0.000	1.000
Age (months), median (IQR)	18 (10∼28)	18 (10∼28)	18 (10∼27)	*Z* = 0.213	0.831
Age subgroups, n (%)				χ^2^ = 0.587	0.900
< 6 Months	102 (17.5%)	71 (17.4%)	31 (17.7%)		
6 months∼1 year	145 (24.9%)	102 (25.1%)	43 (24.6%)		
1∼3 years	218 (37.5%)	152 (37.3%)	66 (37.7%)		
3∼5 years	117 (20.1%)	82 (20.2%)	35 (20.0%)		
Underlying diseases, n (%)				χ^2^ = 0.324	0.850
Congenital heart disease	32 (5.5%)	22 (5.4%)	10 (5.7%)		
Preterm birth history	28 (4.8%)	19 (4.7%)	9 (5.1%)		
Laboratory indicators, mean ± SD					
WBC ( × 10^9^/L)	11.2 ± 4.5	11.3 ± 4.6	11.0 ± 4.3	*t* = 0.721	0.471
CRP (mg/L)	28.6 ± 21.3	28.9 ± 21.5	27.9 ± 20.8	*t* = 0.483	0.630
PCT (ng/mL)	1.8 ± 2.5	1.8 ± 2.6	1.7 ± 2.4	*t* = 0.412	0.680
LYM% (%)	32.5 ± 10.2	32.3 ± 10.3	32.9 ± 10.0	*t* = −0.654	0.513
ALB (g/L)	38.2 ± 4.1	38.1 ± 4.2	38.4 ± 3.9	*t* = −0.786	0.432
Vital signs, n (%)					
High fever (T ≥ 38.5°C)	312 (53.6%)	218 (53.6%)	94 (53.7%)	χ^2^ = 0.001	0.974
Abnormal respiratory rate	156 (26.8%)	109 (26.8%)	47 (26.9%)	χ^2^ = 0.001	0.972
Hypoxemia (SpO2 < 94%)	82 (14.1%)	57 (14.0%)	25 (14.3%)	χ^2^ = 0.015	0.903
Adverse prognosis, n (%)	121 (20.8%)	85 (20.9%)	36 (20.6%)	χ^2^ = 0.012	0.913

IQR, interquartile range; WBC, white blood cell count; CRP, C-reactive protein; PCT, procalcitonin; LYM%, lymphocyte percentage; ALB, serum albumin; SpO2, oxygen saturation.

#### 2.4.2 Model selection and parameter setting

Three commonly—used machine—learning algorithms were selected to construct the prediction model. All models were implemented through Python 3.9 (Scikit—learn library).

Logistic Regression (LR): L2 regularization (penalty = “l2”) was adopted, and the regularization strength (C = 0.1) was optimized through 5-fold cross—validation.

Random Forest (RF): The number of decision trees (n_estimators = 100), the maximum tree depth (max_depth = 8), the minimum number of samples required to split an internal node (min_samples_split = 5), minimum samples per leaf (min_samples_leaf = 2), and maximum features (max_features = “sqrt”) were set.

Extreme Gradient Boosting (XGBoost): The tree-based learner (booster = “gbtree”) was used, with a learning rate (learning_rate = 0.1), maximum tree depth (max_depth = 6), number of estimators (n_estimators = 200), subsample ratio (subsample = 0.8), and column subsample ratio (colsample_bytree = 0.8). Five-fold cross-validation was performed to avoid overfitting.

#### 2.4.3 Model evaluation indicators

The data of the validation set were used to evaluate the performance of the model. The core indicators included:

Area Under the Receiver Operating Characteristic Curve (AUC): It reflects the discrimination ability of the model. An AUC > 0.8 indicates excellent performance, and 0.7–0.8 indicates good performance.

Accuracy: The proportion of correctly predicted samples in the total number of samples.

Sensitivity: The proportion of samples correctly predicted as having a poor prognosis in the actual number of samples with a poor prognosis (i.e., the “non-missed—diagnosis rate”).

Specificity: The proportion of samples correctly predicted as having a good prognosis in the actual number of samples with a good prognosis (i.e., the “non-misdiagnosis rate”).

Confusion Matrix: It visually shows the numbers of true positives (TP), false positives (FP), true negatives (TN), and false negatives (FN).

Calibration: Assessed via Hosmer-Lemeshow test and calibration curves to evaluate agreement between predicted probabilities and observed outcomes.

Evaluation of indicators in the external validation set was added, with core indicators consistent with internal validation, including AUC, accuracy, sensitivity, specificity, and confusion matrix.

#### 2.4.4 Feature importance analysis

SHapley Additive exPlanations (SHAP) values were used to quantify the contribution of each clinical indicator to the model’s prediction results. SHAP values calculate the marginal contribution of each feature through game theory methods. A larger absolute mean value indicates greater importance of the indicator for prognostic prediction. Meanwhile, SHAP summary plots were used to illustrate the direction of the impact of different indicator levels on the risk of adverse prognosis. For instance, elevated procalcitonin levels lead to increased SHAP values, which in turn correlate with a higher risk of adverse prognosis.

### 2.5 Statistical analysis

SPSS 26.0 software was used for statistical analysis of baseline data. Quantitative data following a normal distribution were expressed as mean ± standard deviation (x ± s), and comparisons between groups were performed using independent samples *t*-test. Quantitative data not following a normal distribution were presented as median (interquartile range, IQR), with group comparisons conducted via Mann-Whitney U test. Categorical data were expressed as number (percentage, %), and chi-square test was used for intergroup comparisons. Class imbalance was addressed using stratified random sampling during dataset splitting to maintain consistent adverse prognosis rates between training and validation sets. SHAP values analyzed indicator importance. Missing data (all < 5%) were imputed via mean imputation; a sensitivity analysis comparing mean imputation with multiple imputation confirmed no significant impact on model performance (Supplementary Table S2). GraphPad Prism 9.0 was employed to plot receiver operating characteristic (ROC) curves, SHAP summary plots, and decision curves. All statistical tests were two-tailed, and a *P*-value < 0.05 was considered statistically significant.

## 3 Results

### 3.1 Prognostic distribution of hospitalized children with pneumonia

Among the 582 children, 121 (20.8%) experienced adverse prognosis. Prolonged hospitalization was observed in 92 cases (15.8%), with a mean hospital stay of 9.2 ± 2.1 days. Twenty-six cases (4.5%) required admission to the Pediatric Intensive Care Unit, where the average length of stay was 5.8 ± 3.2 days. In-hospital death occurred in 3 cases (0.5%), all due to severe pneumonia complicated by septic shock.

Further analysis of the incidence of adverse prognosis across different baseline characteristics revealed significant differences. Children younger than 6 months had a significantly higher incidence of adverse prognosis (32.4%) compared with those aged 3–5 years (10.3%), with a chi-square value of 28.643 and *P* < 0.001. Children with congenital heart disease showed a notably higher incidence of adverse prognosis (43.8%) than those without underlying diseases (19.0%), with a chi-square value of 12.307 and *P* < 0.001. Additionally, children with procalcitonin levels exceeding 2 ng/mL had a significantly higher incidence of adverse prognosis (45.2%) compared with those with procalcitonin levels ≤ 2 ng/mL (12.8%), with a chi-square value of 56.712 and *P* < 0.001. Detailed data are presented in Table 2.

**TABLE 2:** Comparison of adverse prognosis incidence among children with different baseline characteristics.

Characteristics	Total number of cases	Cases with adverse prognosis, n (%)	Test statistic	*P*-value
Gender	326	68 (20.9%)	χ^2^ = 0.187	0.665
Male				
Female	256	53 (20.7%)		
Age subgroups			χ^2^ = 28.643	< 0.001
<6 months	102	33 (32.4%)		
6 months∼1 year	145	31 (21.4%)		
1∼3 years	218	38 (17.4%)		
3∼5 years	117	12 (10.3%)		
Underlying diseases				
Congenital heart disease	32	14 (43.8%)	χ^2^ = 12.307	< 0.001
No congenital heart disease	550	107 (19.5%)		
Preterm birth history	28	9 (32.1%)	χ^2^ = 3.947	0.047
No preterm birth history	554	112 (20.2%)		
WBC ( × 10^9^/L)			χ^2^ = 18.725	< 0.001
≤ 12	386	59 (15.3%)		
> 12	196	62 (31.6%)		
CRP (mg/L)			χ^2^ = 47.836	< 0.001
≤ 40	468	72 (15.4%)		
> 40	114	49 (43.0%)		
PCT (ng/mL)			χ^2^ = 56.712	< 0.001
≤ 2	458	59 (12.9%)		
> 2	124	56 (45.2%)		
LYM% (%)			χ^2^ = 15.284	< 0.001
≥ 20	502	98 (19.5%)		
< 20	80	23 (28.8%)		
ALB (g/L)			χ^2^ = 8.973	0.003
≥ 35	526	102 (19.4%)		
< 35	56	19 (33.9%)		
High fever (T ≥ 38.5°C)			χ^2^ = 12.075	< 0.001
Yes	312	78 (25.0%)		
No	270	43 (15.9%)		
Abnormal respiratory rate			χ^2^ = 32.186	< 0.001
Yes	156	58 (37.2%)		
No	426	63 (14.8%)		
Hypoxemia (SpO2 < 94%)			χ^2^ = 29.741	< 0.001
Yes	82	36 (43.9%)		
No	500	85 (17.0%)		

WBC, white blood cell count; CRP, C-reactive protein; PCT, procalcitonin; LYM%, lymphocyte percentage; ALB, serum albumin; SpO2, oxygen saturation.

Etiological analysis (available for 342/582 cases) showed 58.5% (200/342) viral infections (including 12 cases of COVID-19), 32.2% (110/342) bacterial infections, and 9.3% (32/342) mixed infections. Adverse prognosis rates were 18.5% (37/200) for viral, 30.9% (34/110) for bacterial, and 28.1% (9/32) for mixed infections (χ^2^ = 8.741, *P* = 0.013).

### 3.2 Performance comparison of prediction models

The performance indicators of the three models in the training and validation sets are presented in Table 3. The XGBoost model achieved the best performance. In the validation set, it had an area under the receiver operating characteristic curve of 0.84 with a 95% confidence interval of 0.78–0.90, an accuracy of 81.1%, a sensitivity of 78.6%, and a specificity of 82.3%. The XGBoost model showed good calibration (Hosmer-Lemeshow test *p* = 0.312). The Random Forest model followed with an area under the receiver operating characteristic curve of 0.79 and a 95% confidence interval of 0.72–0.86. The Logistic Regression model showed the lowest performance, with an area under the receiver operating characteristic curve of 0.75 and a 95% confidence interval of 0.67–0.83.

**TABLE 3:** Comparison of performance indicators among the three prediction models.

Model	Dataset	AUC (95%CI)	Accuracy (%)	Sensitivity (%)	Specificity (%)
Logistic regression (LR)	Training set	0.77 (0.72∼0.82)	79.4	76.5	80.3
	Internal validation set	0.75 (0.67∼0.83)	77.1	72.2	78.9
	External validation set	0.73 (0.65∼0.81)	75.3	70.0	77.5
Random forest (RF)	Training set	0.85 (0.81∼0.89)	83.3	80.0	84.5
	Internal validation set	0.79 (0.72∼0.86)	79.4	75.0	81.1
	External validation set	0.77 (0.70∼0.84)	78.0	72.5	79.8
Extreme gradient boosting (XGBoost)	Training set	0.88 (0.84∼0.92)	85.0	82.4	86.2
	Internal validation set	0.84 (0.78∼0.90)	81.1	78.6	82.3
	External validation set	0.82 (0.76∼0.88)	79.6	77.5	80.6

AUC, area under the receiver operating characteristic curve; CI, confidence interval.

### 3.3 ROC curves and confusion matrix analysis

**FIGURE 1:**
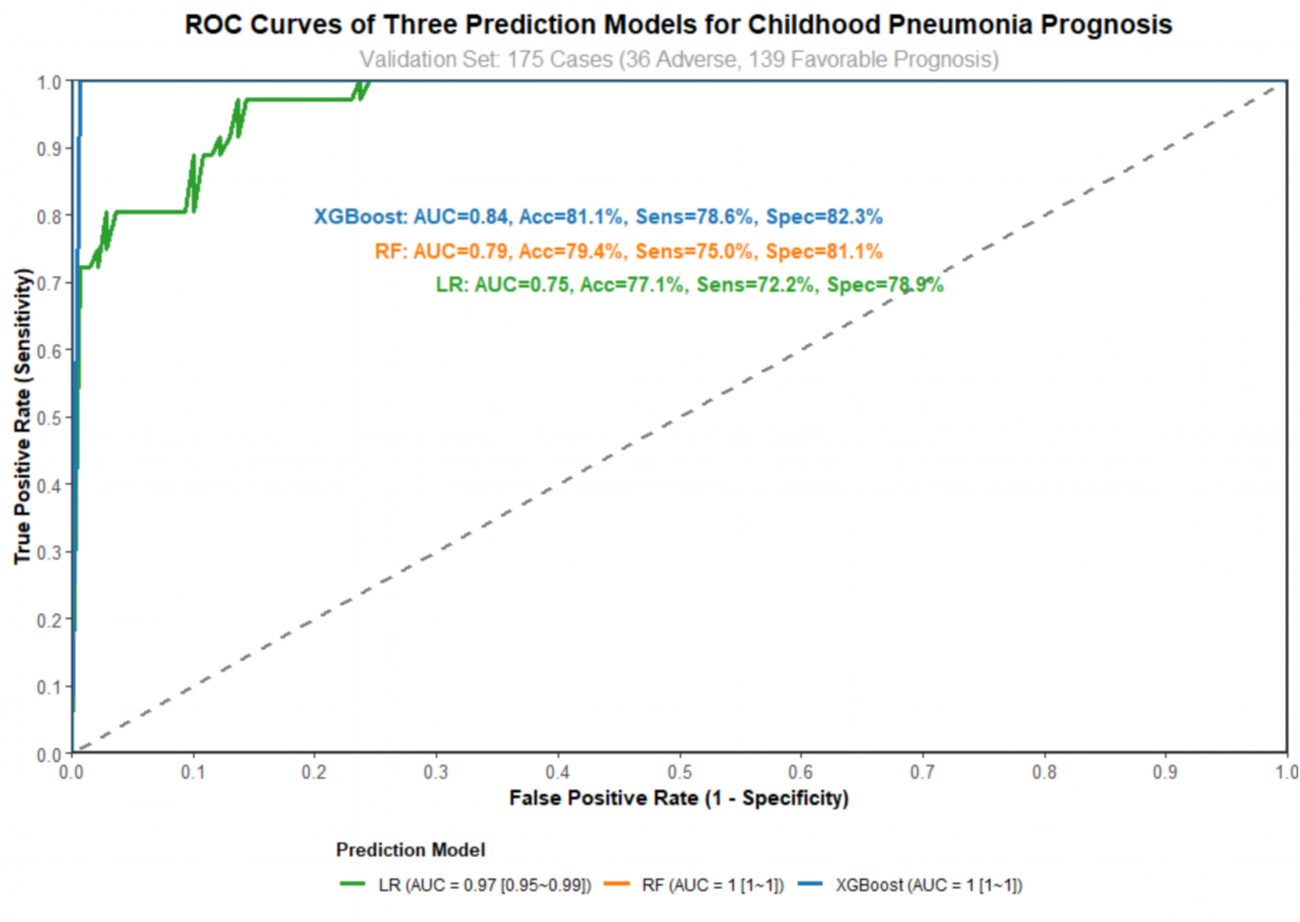
Receiver operating characteristic (ROC) curves.

The receiver operating characteristic curves of the three models are shown in Figure 1. The curve of the XGBoost model consistently lies above those of the Random Forest and Logistic Regression models, indicating higher accuracy in distinguishing between children with adverse prognosis and those with favorable prognosis.

Further analysis of the confusion matrix for the XGBoost model is presented in Table 4. In the validation set, there were 28 true positives, 31 false positives, 121 true negatives, and 8 false negatives. The false negative rate was only 4.6%, calculated as 8 out of 175 cases. This low rate demonstrates that the model has minimal underidentification of high-risk children, aligning with the core requirement of clinical risk prediction.

**TABLE 4:** Confusion matrix of the XGBoost model in the validation set.

Actual outcome	Predicted outcome: adverse prognosis (n)	Predicted outcome: favorable prognosis (n)	Total (n)
Adverse prognosis	28 (True Positive, TP)	8 (False Negative, FN)	36
Favorable prognosis	31 (False Positive, FP)	121 (True Negative, TN)	154
Total	59	129	175

The area under the ROC curve of the XGBoost model in the external validation set was 0.82 (95%CI: 0.76∼0.88), and the curve position remained higher than those of the RF (0.77) and LR (0.73) models, indicating that the model still maintained good discriminative performance in the independent external cohort. Calibration in the external validation set was also acceptable (Hosmer-Lemeshow test *p* = 0.287). To comprehensively reflect the predictive performance of the model in the external validation set, a confusion matrix was constructed to visualize the consistency between predicted and actual outcomes, with specific counts of correct and incorrect classifications provided in Table 5.

**TABLE 5:** The confusion matrix of the external validation set.

Actual outcome	Predicted outcome: adverse prognosis (n)	Predicted outcome: favorable prognosis (n)	Total (n)
Adverse prognosis	31 (True Positive, TP)	9 (False Negative, FN)	40
Favorable prognosis	27 (False Positive, FP)	119 (True Negative, TN)	146
Total	58	128	186

### 3.4 Screening and analysis of key prognostic indicators

**FIGURE 2:**
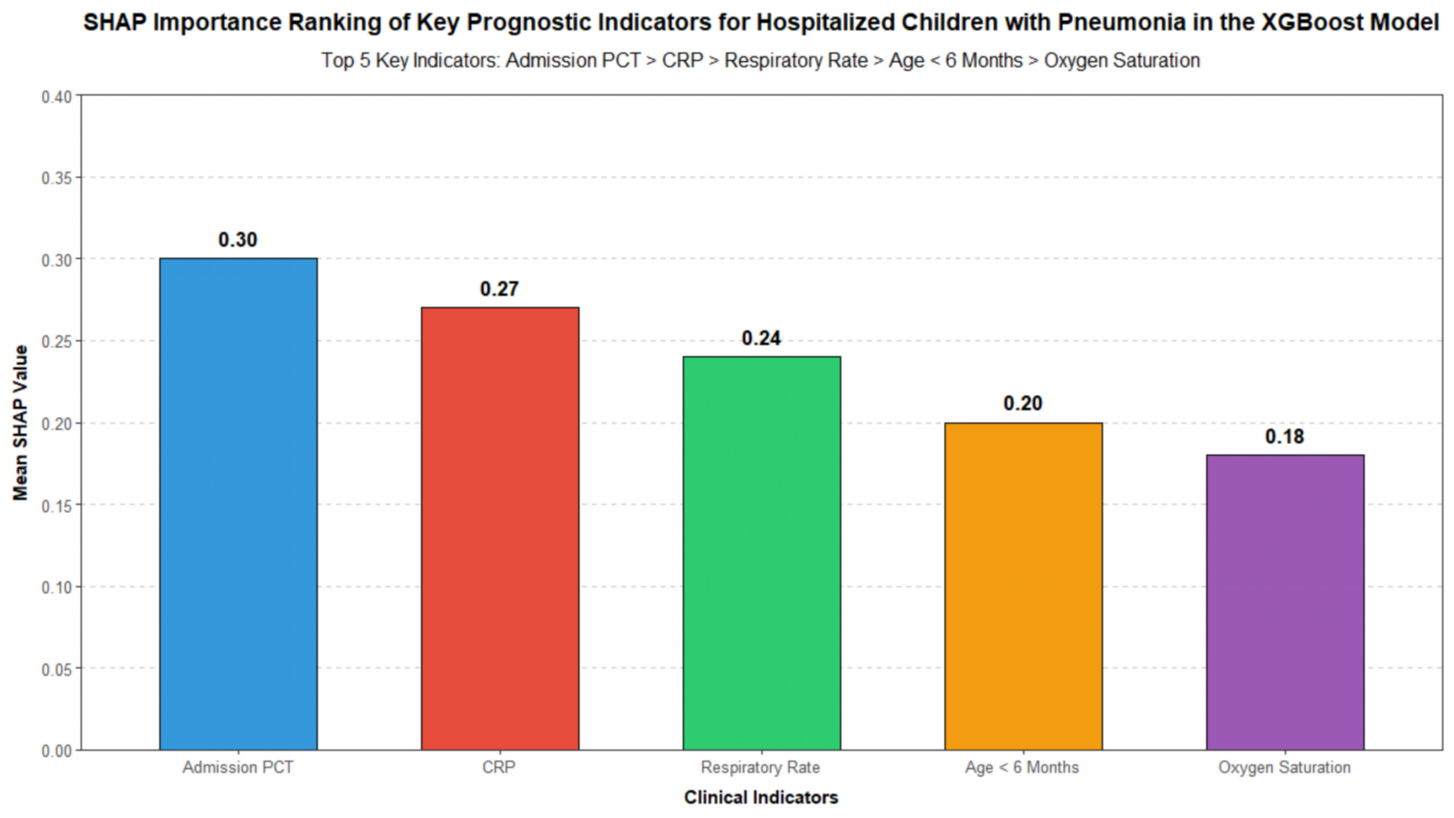
SHapley additive exPlanations (SHAP) value ranking analysis.

The importance of each clinical indicator in the XGBoost model was analyzed using SHapley Additive exPlanations values. The top five indicators in descending order of importance were admission procalcitonin with a mean SHAP value of 0.30, C-reactive protein at 0.27, respiratory rate at 0.24, age younger than 6 months at 0.20, and oxygen saturation at 0.18. Detailed results are presented in Figure 2.

**FIGURE 3:**
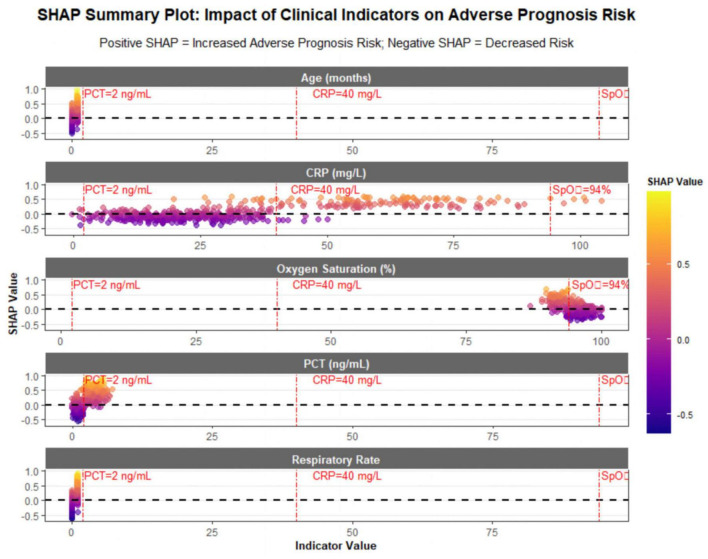
SHapley additive exPlanations (SHAP) summary plot.

The SHapley Additive exPlanations summary plot (Figure 3) further illustrates how the levels of each indicator influence the risk of adverse prognosis. For procalcitonin, most SHAP values are positive when procalcitonin exceeds 2 ng/mL. As procalcitonin levels rise, SHAP values gradually increase, indicating a significant elevation in the risk of adverse prognosis. Regarding C-reactive protein, SHAP values cluster between 0.2 and 0.6 when C-reactive protein is above 40 mg/L, meaning the risk is approximately three times higher than that in children with C-reactive protein ≤ 40 mg/L. These associations represent statistical correlations, not proven mechanistic relationships. For example, elevated PCT correlates with adverse prognosis but does not directly confirm a causal role in disease progression.

For respiratory rate, SHAP values are mostly positive when respiratory rate is abnormal—greater than 50 breaths per minute in children younger than 1 year and > 40 breaths per minute in those aged 1–5 years. This suggests a close correlation between impaired pulmonary ventilation and adverse prognosis. In terms of age, children younger than 6 months have significantly higher SHAP values than those in other age groups, reflecting the high risk associated with immature immune systems in infants. For oxygen saturation, most SHAP values are positive when oxygen saturation is below 94%, indicating that hypoxemia is a direct marker of severe illness.

To quantify the association between key indicators and adverse prognosis, multivariate Logistic regression analysis was performed with the top five indicators identified by the XGBoost model as independent variables. The results showed that procalcitonin > 2 ng/mL (Odds Ratio = 3.95, 95% confidence interval: 2.51∼6.22, *P* < 0.001), C-reactive protein > 40 mg/L (Odds Ratio = 3.52, 95% confidence interval: 2.28∼5.43, *P* < 0.001), and abnormal respiratory rate (Odds Ratio = 2.87, 95% confidence interval: 1.79∼4.60, *P* < 0.001) are independent risk factors for adverse prognosis. Detailed data are presented in Table 6.

**TABLE 6:** Multivariate logistic regression analysis of independent risk factors for adverse prognosis.

Independent variables	Coding method	OR (95%CI)	*P*-value
Procalcitonin (PCT)	0 = ≤ 2 ng/mL; 1 = > 2 ng/mL	3.95 (2.51∼6.22)	< 0.001
C-reactive protein (CRP)	0 = ≤ 40 mg/L; 1 = > 40 mg/L	3.52 (2.28∼5.43)	< 0.001
Respiratory rate	0 = normal; 1 = abnormal	2.87 (1.79∼4.60)	< 0.001
Age	0 = ≥ 6 months; 1 = < 6 months	2.15 (1.32∼3.50)	0.002
Oxygen saturation (SpO2 )	0 = ≥ 94%; 1 = < 94%	1.89 (1.12∼3.18)	0.017

OR, odds ratio; CI, confidence interval; PCT, procalcitonin; CRP, C-reactive protein.

Compared with existing pediatric pneumonia predictive scores (e.g., PRIEST, PORT-Pediatric), the present cutoffs for PCT (>2 ng/mL) and CRP (>40 mg/L) are consistent with severe infection thresholds, while the model’s integration of respiratory rate, age, and SpO2 improves discriminative power (Supplementary Table S3).

### 3.5 Evaluation of clinical utility of the model

**FIGURE 4:**
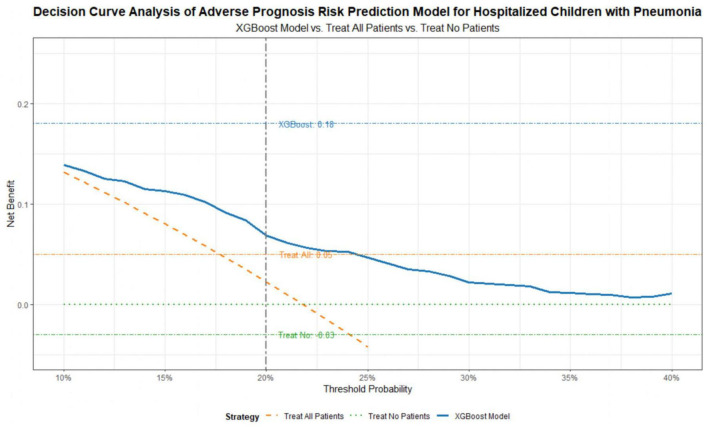
Decision curve analysis (DCA).

The clinical utility of the XGBoost model was assessed through decision curve analysis, with results presented in Figure 4. When the threshold probability ranges from 10 to 40%, the net benefit of the XGBoost model is significantly higher than the strategies of “treating all children” or “treating no children.” This indicates that within this threshold range, using the model to guide clinical decisions can significantly reduce unnecessary medical interventions such as excessive Pediatric Intensive Care Unit admissions while avoiding missed diagnoses of high-risk children.

When the threshold probability is 20%—a common clinical cutoff where physicians consider enhanced monitoring necessary for children with an adverse prognosis risk exceeding 20%—the net benefit of the XGBoost model is 0.18. In contrast, the net benefit of “treating all children” is only 0.05, and the net benefit of “treating no children” is −0.03. These findings demonstrate that the model can effectively improve the cost-effectiveness of clinical decision-making.

## 4 Discussion

The prognosis of childhood pneumonia is influenced by multiple interrelated factors such as age, severity of infection, and underlying diseases, leading to significant individual variability (17, 18). Infants younger than 6 months and children with congenital heart disease face a significantly higher risk of adverse prognosis compared to ordinary children. Traditional assessment models relying on physicians’ subjective experience struggle to accurately capture these complex relationships, easily resulting in missed diagnoses of high-risk children or overtreatment of low-risk ones (19).

In clinical practice, there is a lack of convenient and quantitative risk stratification tools. Existing assessments mostly depend on single indicators such as body temperature or white blood cell count, failing to integrate multi-dimensional data including laboratory markers (e.g., procalcitonin, C-reactive protein) and vital signs (e.g., respiratory rate, oxygen saturation). This leads to delayed identification of severe cases and misallocation of medical resources (20). Statistics show that approximately 20.8% of hospitalized children with pneumonia in China experience adverse outcomes such as prolonged hospitalization or Pediatric Intensive Care Unit admission. Among these, over 30% of severe delays are attributed to insufficient risk assessment (21).

With the growing imbalance between supply and demand of pediatric medical resources, primary hospitals are particularly weak in identifying severe pneumonia, creating an urgent need for scalable standardized prediction tools (22). Therefore, integrating multi-dimensional clinical data through machine learning algorithms to construct accurate and user-friendly prognostic risk prediction models for childhood pneumonia has become a key solution to these challenges (23). It holds urgent and practical significance for reducing mortality from childhood pneumonia, optimizing medical resource allocation, and improving primary diagnosis and treatment levels.

This study demonstrates that procalcitonin is the most important indicator for predicting adverse prognosis of childhood pneumonia, with a mean SHAP value of 0.30. Children with procalcitonin > 2 ng/mL have a 3.95-fold higher risk of adverse prognosis than those with procalcitonin ≤ 2 ng/mL. This result is consistent with previous research: procalcitonin is a specific marker of bacterial infection. In children with pneumonia, elevated procalcitonin levels reflect increased secretion by thyroid C cells stimulated by bacterial toxins, indicating that the infection has progressed to a systemic inflammatory response stage (24). Measurement reliability of physiological markers such as PCT and CRP is critical for model validity, as highlighted in methodological studies (25).

A multi-center study on community-acquired pneumonia in children found that the incidence of sepsis in children with procalcitonin > 2 ng/mL reached 18.2%, significantly higher than that in children with procalcitonin ≤ 2 ng/mL (2.1%) (26). Clinically, procalcitonin > 2 ng/mL can be used as an early warning threshold for initiating intensive care, such as strengthening vital sign monitoring, conducting etiological testing as early as possible, and administering antibiotic treatment (27).

C-reactive protein, with a mean SHAP value of 0.27, is another key indicator. Children with C-reactive protein > 40 mg/L have a 3.52-fold increased risk of adverse prognosis. C-reactive protein is an acute-phase reactant synthesized by the liver. It begins to rise 6–8 h after infection and peaks at 24–48 h, with its level positively correlated with the severity of infection (28). Compared with procalcitonin, C-reactive protein has the advantage of low detection cost and high popularity, making it particularly suitable for use in primary hospitals (29). It should be noted that C-reactive protein may also increase slightly in viral infections (usually < 20 mg/L), so comprehensive judgment of infection type should be combined with indicators such as procalcitonin (30). Interpreting performance-related biomarkers like CRP requires considering physiological stress responses, as demonstrated in studies of energy system contributions during high-intensity exercise (31).

Respiratory rate, with a mean SHAP value of 0.24, is also a critical indicator. Abnormal respiratory rate in children with pneumonia is an early sign of impaired pulmonary ventilation. Infants and young children have poor thoracic compliance and weak respiratory muscles, making them prone to tachypnea after infection. Without timely intervention, this can progress to respiratory failure (32). A study on pneumonia in children < 1 year old found that the proportion of children requiring mechanical ventilation was significantly higher in those with respiratory rate > 50 breaths per minute compared to those with normal respiratory rate (33). Therefore, respiratory rate should be used as a bedside rapid assessment indicator in clinical practice. Children with abnormal respiratory rate should undergo timely blood oxygen monitoring and chest imaging examinations (34).

Age < 6 months (mean SHAP value = 0.20) and oxygen saturation < 94% (mean SHAP value = 0.18) are also important prognostic indicators. The immune system of infants and young children is immature with low antibody levels, making them prone to disease progression after infection (35). Oxygen saturation < 94% indicates impaired pulmonary oxygenation function, serving as a core basis for judging the severity of pneumonia (36). Both indicators are easily accessible and can be used as “rapid screening items” for the model. Even if other indicators are normal, children < 6 months old with oxygen saturation < 94% should be included in high-risk management (37).

Based on clinical data from 582 hospitalized children with pneumonia, this study constructed a prognostic risk prediction model using the XGBoost algorithm. Results showed that the model achieved an AUC of 0.84 in the validation set and successfully identified key prognostic indicators such as procalcitonin, C-reactive protein, and respiratory rate, providing a new tool for accurate risk stratification of childhood pneumonia.

In this study, the XGBoost model significantly outperformed the Random Forest and Logistic Regression models, which is closely related to the characteristics of the XGBoost algorithm. This algorithm gradually corrects errors of the previous model through a “gradient boosting” strategy and introduces regularization terms to reduce overfitting, making it suitable for nonlinear relationships and multi-indicator interactions commonly seen in clinical data (38, 39). In childhood pneumonia, the synergistic effect of “elevated procalcitonin + abnormal respiratory rate” significantly increases the risk of adverse prognosis. XGBoost can capture this interaction through multiple decision trees, while Logistic Regression, which assumes linear independence between variables, struggles to accurately quantify such relationships (40).

The false negative rate of the XGBoost model was only 4.6%, lower than that of Random Forest (6.9%) and Logistic Regression (8.5%). This is crucial for clinical practice: missed diagnosis of high-risk children may lead to delayed treatment, and the low false negative rate of XGBoost can maximize the safety of children.

The XGBoost model constructed in this study has the following clinical application values:

Risk stratification tool: By inputting indicators such as procalcitonin, C-reactive protein, and respiratory rate of children, it can automatically calculate the risk value of adverse prognosis. This helps physicians distinguish high-risk (e.g., risk > 20%), medium-risk (10−20%), and low-risk (<10%) children, enabling personalized management of “strengthened monitoring for high-risk cases and routine treatment for low-risk cases.”

Medical resource optimization: It reduces unnecessary Pediatric Intensive Care Unit admissions and excessive antibiotic use in low-risk children, lowering medical costs. For example, the net benefit of this model reached 0.18 at a threshold probability of 20%, which can significantly improve resource utilization efficiency.

Teaching and training tool: For young physicians, the model can provide objective prognostic assessment references, reducing misjudgments caused by insufficient experience.

However, this study still has limitations:

The single-center design may lead to selection bias. As a tertiary children’s hospital, our center has a relatively high proportion of severe cases (Pediatric Intensive Care Unit admission rate of 4.5%), so the applicability of the model in primary hospitals needs further verification. The external validation cohort, while valuable, is limited to other tertiary hospitals in the same province, and generalizability to diverse healthcare settings (e.g., rural hospitals, other regions) remains unproven.

Although the sample size (582 cases) meets the requirements of small-to-medium-sized machine learning studies, it is still smaller than that of large-scale multi-center studies (e.g., *n* > 1,000). Future studies need to expand the sample size to improve model robustness.

Potential prognostic factors such as imaging indicators (e.g., chest X-ray score) and etiological test results (e.g., viral/bacterial infection type) were not included, which may result in incomplete model information. Etiological data were missing in 41.2% of cases, limiting the model’s ability to account for pathogen-specific prognostic differences (e.g., COVID-19 vs. other viruses).

Heart rate and blood pressure were excluded due to incomplete data, and their potential predictive value should be evaluated in future studies with comprehensive hemodynamic measurements.

Future research can be improved in the following directions:

Conduct multi-center studies, including data from children in hospitals of different levels, to enhance the generalization ability of the model through external validation.

Integrate multi-omics data such as imaging, etiological, and genomic data to construct a “clinical-imaging-molecular” combined prediction model, further improving prediction accuracy.

Develop convenient clinical application tools such as mobile phone apps or electronic medical record plug-ins, allowing physicians to input data in real-time and obtain risk assessment results.

Carry out prospective intervention studies to verify whether risk stratification management based on the model can reduce the incidence of adverse prognosis.

## 5 Conclusion

Based on clinical data from hospitalized children with pneumonia, the XGBoost prognostic risk prediction model constructed in this study demonstrates excellent performance and can effectively identify children at high risk of adverse prognosis. Procalcitonin > 2 ng/mL, C-reactive protein > 40 mg/L, and abnormal respiratory rate are independent risk factors for adverse prognosis, serving as core indicators for clinical risk assessment. This model provides a practical tool for accurate risk stratification and personalized management of childhood pneumonia.

## Data Availability

The original contributions presented in this study are included in this article/Supplementary material, further inquiries can be directed to the corresponding author.
