# Supplementary material for: Exploration of the correlation between clinical indicators and prognosis in hospitalized children with pneumonia and construction of a risk prediction model based on machine learning algorithms
Source: Front Med (Lausanne). 2026 Jan 28;13:1747935. doi: 10.3389/fmed.2026.1747935 (PMC12891092; doi:10.3389/fmed.2026.1747935)
Supplement: Supplementary file 1 [file Table_1.docx]

**Supplementary Table S1: Sensitivity Analysis of Adverse Prognosis Definitions**

| **Adverse Prognosis Definition** | **Model** | **Dataset** | **AUC (95%CI)** | **Accuracy (%)** | **Sensitivity (%)** | **Specificity (%)** | **False Negative Rate (%)** |
| --- | --- | --- | --- | --- | --- | --- | --- |
| Composite (Prolonged Hospitalization >7 days + PICU Admission + In-hospital Death) | XGBoost | Internal Validation Set | 0.84 (0.78~0.90) | 81.1 | 78.6 | 82.3 | 4.6 |
|  |  | External Validation Set | 0.82 (0.76~0.88) | 79.6 | 77.5 | 80.6 | 4.8 |
| Severe Only (PICU Admission + In-hospital Death) | XGBoost | Internal Validation Set | 0.87 (0.81~0.93) | 88.0 | 83.3 | 88.3 | 2.3 |
|  |  | External Validation Set | 0.85 (0.79~0.91) | 86.5 | 81.2 | 87.0 | 2.6 |
| Prolonged Hospitalization >10 days + PICU Admission + In-hospital Death | XGBoost | Internal Validation Set | 0.85 (0.79~0.91) | 83.4 | 80.1 | 84.4 | 3.4 |
|  |  | External Validation Set | 0.83 (0.77~0.89) | 82.2 | 78.8 | 83.0 | 3.7 |

Note: Severe-only definition excludes prolonged hospitalization (>7 days) to focus on life-threatening outcomes. Prolonged hospitalization >10 days uses a stricter threshold for persistent illness.

**Supplementary Table S2: Comparison of Model Performance Between Mean Imputation and Multiple Imputation**

| **Model** | **Imputation Method** | **Dataset** | **AUC (95%CI)** | **Accuracy (%)** | **Sensitivity (%)** | **Specificity (%)** | **Hosmer-Lemeshow Test (p-value)** |
| --- | --- | --- | --- | --- | --- | --- | --- |
| XGBoost | Mean Imputation | Internal Validation Set | 0.84 (0.78~0.90) | 81.1 | 78.6 | 82.3 | 0.312 |
|  | Multiple Imputation (5 iterations) | Internal Validation Set | 0.83 (0.77~0.89) | 80.6 | 77.2 | 81.8 | 0.345 |
|  | Mean Imputation | External Validation Set | 0.82 (0.76~0.88) | 79.6 | 77.5 | 80.6 | 0.287 |
|  | Multiple Imputation (5 iterations) | External Validation Set | 0.81 (0.75~0.87) | 79.0 | 76.3 | 80.1 | 0.309 |
| Random Forest | Mean Imputation | Internal Validation Set | 0.79 (0.72~0.86) | 79.4 | 75.0 | 81.1 | 0.276 |
|  | Multiple Imputation (5 iterations) | Internal Validation Set | 0.78 (0.71~0.85) | 78.9 | 73.9 | 80.5 | 0.298 |
| Logistic Regression | Mean Imputation | Internal Validation Set | 0.75 (0.67~0.83) | 77.1 | 72.2 | 78.9 | 0.241 |
|  | Multiple Imputation (5 iterations) | Internal Validation Set | 0.74 (0.66~0.82) | 76.6 | 71.1 | 78.3 | 0.263 |

Note: Multiple imputation was performed with 5 iterations using the MICE algorithm. No statistically significant differences in performance were observed between imputation methods (all p>0.05).

**Supplementary Table S3: Comparison of Cutoff Values with Existing Pediatric Pneumonia Predictive Scores**

| **Predictive Score** | **Key Indicators** | **Cutoff Values** | **Target Population** | **AUC (Reported)** | **Study Design** |
| --- | --- | --- | --- | --- | --- |
| Present Study (XGBoost Model) | PCT  CRP  Respiratory Rate  Age  SpO₂ | PCT >2 ng/mL  CRP >40 mg/L  Abnormal (age-specific)  <6 months  <94% | Hospitalized children with CAP (1 month–5 years) | 0.84 (Internal)  0.82 (External) | Retrospective, single-center + external validation (Shaanxi, China) |
| PRIEST Score | Age  Temperature  Respiratory Rate  Oxygen Saturation  CRP | <2 years  ≥38.5°C  Abnormal (age-specific)  <95%  >60 mg/L | Emergency department children with CAP (3 months–16 years) | 0.81 | Prospective, multicenter (Europe) |
| PORT-Pediatric Score | Age  Temperature  Respiratory Rate  WBC  Comorbidities | <2 years  ≥38.3°C  Abnormal (age-specific)  >15×10⁹/L  Yes (e.g., heart disease) | Outpatient/inpatient children with CAP (2 months–18 years) | 0.78 | Retrospective, multicenter (USA) |
| CAP-Pedi Score | Age  Respiratory Distress  SpO₂  PCT  Comorbidities | <1 year  Yes  <94%  >1 ng/mL  Yes (e.g., preterm birth) | Hospitalized children with CAP (1 month–5 years) | 0.83 | Prospective, single-center (China) |
| WHO Pneumonia Severity Criteria | Respiratory Rate  Chest Indrawing  SpO₂ | Abnormal (age-specific)  Yes  <93% | Children with pneumonia (2 months–5 years) | 0.76 | Cross-sectional, multicenter (Global) |

Note: CAP = community-acquired pneumonia; SpO₂ = oxygen saturation; WBC = white blood cell count.
